# Sensorial, Melissopalynological and Physico-Chemical Characteristics of Honey from Babors Kabylia’s Region (Algeria)

**DOI:** 10.3390/foods10020225

**Published:** 2021-01-22

**Authors:** Asma Ghorab, María Shantal Rodríguez-Flores, Rifka Nakib, Olga Escuredo, Latifa Haderbache, Farid Bekdouche, María Carmen Seijo

**Affiliations:** 1 Laboratoire d’Ecologie et Environnement, Faculté des Sciences de la Nature et de la Vie, Université A. Mira de Bejaia, Bejaia 06000, Algeria; 2 Department of Vegetal Biology and Soil Sciences, Facultade de Ciencias, Universidade de Vigo, 32004 Ourense, Spain; mariasharodriguez@uvigo.es (M.S.R.-F.); nakib.rifka@gmail.com (R.N.); oescuredo@uvigo.es (O.E.); 3 Laboratory of Food Quality and Food Safety, University of Mouloud Mammeri, Tizi Ouzou 15000, Algeria; 4 Research Laboratory in Food Technology (LRTA), M’hamed Bougara University, Avenue de l’indépendance, Boumerdes 35000, Algeria; l.haderbache@univ-boumerdes.dz; 5 Department of Ecology and Environment, FSNV, University of Batna 2, Batna 05000, Algeria; bekdouche_21@yahoo.fr

**Keywords:** honey, Babors Kabylia, sensorial properties, melissopalynology, quality parameters, multivariate analysis

## Abstract

This study aimed to characterize the honeys of Babors Kabylia through sensory, melissopalynological and physico-chemical parameters. Thirty samples of honey produced in this region were collected over a period of two years and analyzed. All the samples presented physico-chemical parameters in conformity with legislation on honey quality, with few exceptions, linked mainly to beekeeping management. The pollen spectrum revealed a great diversity with 96 pollen types. The main pollen types were spontaneous species as Fabaceae (*Hedysarum*, *Trifolium*, Genisteae plants), Asteraceae plants, Ericaceae (*Erica arborea* L.) or *Myrtus* and *Pistacia*. The sensory properties of samples showed a high tendency to crystallization, the colors were from white to brown, but most of them had gold color. Smell and odor corresponded mainly to vegetal and fruity families and in taste perceptions besides sweetness highlighted sourness and saltiness notes. Seventeen samples were polyfloral, one was from honeydew and twelve were monofloral from heather, genista plants, sulla, blackberry or Asteraceae. Heather and the honeydew samples showed the darkest color, the highest electrical conductivity and phenol and flavonoid content. A statistical analysis based on the most representative pollen types, sensory properties and some physico-chemical components allowed the differentiation of honey samples in terms of botanical origin.

## 1. Introduction

Honey is one of the apian products more linked to the territory in which was produced, due to plant communities of the area, climate, soil and apicultural practices drive its characteristics. In Algeria, beekeeping is considered an integral part of the agricultural and rural routine. It is practiced in several regions but has been more important in the north of the country thanks to the appropriate climatic conditions and the great floristic biodiversity that provides honey resources during most of the year [1]. There are more than 20,000 beekeepers with 700,000 hives throughout Algeria, mainly are modern hives (Langstroth type and lesser Dadant type) and rarely are traditional hives. About 90% are independent and amateur and only 10% are professionals. 

Babors Kabylia’s region is situated at the North east of Algeria being one of the most interesting regions for honey production in bio-geographical terms [2]. It has been considered as a biodiversity hotspot because of the richness of its flora and the presence of high number of endemic plants [3]. Within this large plant biodiversity, melliferous plants constitute an important part, so that it is possible to produce a wide variety of honey types [4,5,6,7]. According to [8,9], the plant species visited by the bees as well as the environment in which the honey was produced seem to have a strong influence on its quality and quantity; hence, it is possible to relate it to its geographical origin and on the other hand, honey could be a footprint of its environment.

The tellien sector of the Babors is made up of folded and scaled units. The soils are of a shisty and marly calcareous nature. Its Mediterranean-type climate is characterized by a rainy season mainly consisting of thunderstorms and torrential rains, concentrated during a very wet period from October to March with an average annual rainfall of nearly 900 mm/year, and a dry season between June and September. The vegetation is characterized by woodlands and shrubs, spontaneous plants, agricultural fields and other grasslands and hedgerows, forming a relatively heterogeneous landscape with numerous forage opportunities for honeybees. 

The relief is from the sea level to high mountains (more than 1200 m). At the north slopes of these mountains, the forest vegetation is very dense with woodlands of resinous species such as *Cedrus atlantica* (Endl.) Carrière and *Abies numidica* de Lannoy ex Carrière. and caducifolia oaks such as *Quercus canariensis* Willd. and *Quercus afares* Pomel. The southern slopes are practically devoid of forest vegetation being shrubs such as *Calicotome spinosa* (L.) Link. and some herbaceous plants as *Ampelodesma mauritanicus* (Poir.) Durand & Schinz. At altitudes below 1200 m, appear some degraded green oak forests (*Quercus ilex* L.), but is the domain of cork oak forest (*Quercus suber* L.), with firstly the humid facies of *Cytisus villosus* Pourr. and then, at lower altitudes, the thermophilic facies of *Erica arborea* L. Most of the beekeeping is practiced in this area. The maquis constitutes an interesting plant community for apiculture; more particularly *Erica arborea* and *Pistacia lentiscus* L. association, which covers the slopes located at less than 600 m of altitude in inland regions. It includes, among others, *Cistus salviifolius* L., *Arbutus unedo* L., *Clinopodium vulgare* L., *Lavandula stoechas* L., *Daphne gnidium* L. and *Genista tricuspidata* Desf. Its degradation also promotes a great biodiversity of spontaneous species characteristics of stripped soils: *Cistus monspeliensis* L., *Bellis sylvestris* Cirillo, *Hypochaeris radicata* L., *Hedysarum coronarium* L., *Stachys ocymastrum* (L.) Briq. and Poaceae as *Ampelodesmos mauritanicus*, *Briza maxima* L., *Aira tenorei* Guss., *Festuca coerulescens* Desf. or *Cynosurus echinatus* L. [10].

On the side of Draa El Kaid, between 500 and 700 m of altitude, close to the villages, grows a shrub with *Retama sphaerocarpa* (L.) Boiss., *Calicotome spinosa*, *Thymus munbyanus* subsp. *ciliatus (Desf.) Greuter & Burdet*., *Capparis spinose* L., *Ziziphus lotus* (L.) Lam. and *Teucrium polium* L. On the North, the Gouraya National Park, have a vegetation characterized by a degraded shrub with *Pinus halepensis* Mill. dominated by *Quercus coccifera* L., *Erica arborea*, *Erica multiflora* L., *Stachys ocymastrum* and *Glebionis coronaria* (L.) Spach. [10]. Main agricultural crops are near to the coast and the rivers. The most common are Solanaceae as potatoes, tomatoes, peppers or Brassicaceae as cauliflower crops. Fruit arboriculture is represented by orchards of orange, lemon, apples and sometimes medlar. Deserving a special mention for viticulture are olives and fig trees.

Honey, the fruit of collaboration between the plant and animal worlds, has always been considered a sacred product because of its attributed nutritional and therapeutic benefits [11]. Furthermore, beekeeping is an environmentally friendly practice useful to promote local economy in areas with water scarcity and to facilitate pollination services in highly valued ecosystems. As occurs in many parts of the world, in Algeria, people prefer local beekeeping and consumers get their honey directly from beekeepers, trusting them for the quality and botanical origin of the honey. However, the characteristics of local productions are poorly studied and most Algerian honeys are mislabeled. In this context, increasing knowledge in local honeys contributes to their valorization and to avoid frauds for consumers. One of the main tasks is the authentication of the predominant botanical origin and quality. In this framework, sensory characteristics are the first attributes distinguished for consumers and together with melissopalynology deepen in the botanical and geographical origin of the honey [12]. Physico-chemical parameters complete the information to characterize local productions.

The objective of this study was to investigate the characteristics of the honey produced in one of declared Mediterranean biodiversity hotspots. For this purpose, thirty honey samples collected during the years 2018–2019, in the Babors Kabylia’s region, a large geographical area of Northern Algeria, were analyzed.

## 2. Materials and Methods

### 2.1. Study Area and Honey Samples

The present study was conducted on 30 honey samples obtained from *Apis mellifera intermissa* apiaries situated throughout Babors Kabylia’s region (North East of Algeria). The samples were collected during spring and summer seasons (2018–2019) and the honey extraction from the combs was by centrifugation (Table 1). Then the samples were stored in glass jars at −4 °C until its analysis.

During the harvest season, the main melliferous plants of the region were identified and reference slides of pollen were prepared [13], for comparison with the pollen types found in the honey samples.

The following determinations were carried out: sensorial analysis (color, smell, taste and aroma), melissopalynological analysis and physico-chemical analysis (quality parameters, phenol and flavonoid content and main mineral content).

### 2.2. Sensorial Analysis

Sensory analysis (visual, olfactory and gustatory characteristics) of collected honey samples was performed by a tasting panel constituted of a group of tasters (8 people) previously selected and trained according to international standards. The sensory test was performed in a sensory room under natural white light at room temperature. The samples were presented to the panel as 20 mL in small transparent glasses. Water was provided for rinsing the mouth between samples. Each honey sample was individually evaluated by descriptive grades using scales (1–10). The descriptors used for the evaluation can be seen in Table 2.

### 2.3. Melissopalynological Analysis

Pollen analysis was performed for quantitative results (number of pollen grains per gram of honey) and qualitative results (pollen spectra of the honey samples).

#### 2.3.1. Quantitative

The methodology is based on the methods of melissopalynology [13]. Ten grams of honey were weighed and fully dissolved in 40 mL of warm distilled water (not above 40 °C). The solution was centrifuged for 10 min at 4500 rpm and the supernatant was discarded. Afterwards, 40 mL of distilled water was added prior to centrifugation for 5 min. The supernatant was again discarded until a volume of 5 mL and then the sediment was vortexed. For the microscopical analysis two drops (10 µL) of this sediment were deposited in separate, over a slide. The total number of pollen grains in each drop were counted and the results were expressed as number of pollen grains per g of honey considering the mean value of both drops. Honeys are grouped considering the number of pollen grains per gram of honey (PG/G) into one of the following classes: Class I with less than 2000 pollen grains; Class II with 2000 to 10,000 pollen grains; Class III with 10,000 to 50,000 pollen grains; Class IV with between 50,000 and 100,000 pollen grains; and Class V with more than 100,000 pollen grains.

#### 2.3.2. Qualitative

The obtained sediment for quantitative analysis was centrifuged again and the supernatant was discarded. After vortexing, two drops (100 µL) of sediment were placed separately on a slide and distributed over an area of about 24 × 24 mm. Examination of pollen slides was performed using an optical microscope (400× or 1000×, as appropriate). The percentage of representation for each type of pollen was calculated by counting at least 500 pollen grains per sample. The pollen grains were classified as pollen type, as genus or a single species, when it was possible. Following the recommendations suggested by [13], the pollen frequency classes were determined.

### 2.4. Physico-Chemical Analysis and Color Determination

Quality parameters including honey freshness (HMF and diastase content), moisture, electrical conductivity, pH and free acidity were determined in duplicate and following methodologies proposed by the International Honey Commission [14].

#### 2.4.1. Honey Freshness

HMF content was determined through the White spectrophotometric method. The absorbance of a honey solution was measured at 284 and 336 nm with a UV-visible spectrophotometer (Thermo Scientific Helios Gamma, Chorley, UK) against a blank. The determination of diastase activity was based on the quantity of starch converted by a honey solution and the absorbance of the resulting blue color. It was determined spectrophotometrically at 660 nm using a UV-visible spectrophotometer (Thermo Scientific Helios Gamma, Chorley, UK) at different times to an end point below 0.235. Diastase activity was expressed in diastase index (DI) Gothe Scale.

#### 2.4.2. Other Quality Parameters

Water content was determined with a Carl-Zeiss Jena refractometer by measuring the refractive indices at 20 °C. The percentage of water was calculated using the CHATAWAY table. Electrical conductivity was performed at 20 °C in a 20% (*w*/*v*) honey solution (dry matter basis) in CO_2_-free deionized distilled water using a portable conductivity meter (Knick Portamess^®^ 913 Conductivity, Beuckestr, Berlin, Germany). The values were expressed in mS/cm. pH was measured by a pHmeter (WTW inoLab pH 750) on a solution containing 10 g of honey dissolved in 75 mL of distilled water; the same solution was titrated for free acidity with 0.1 M sodium hydroxide (NaOH) solution up to a pH of 8.3. The results were expressed in meq/kg.

#### 2.4.3. Color of Honey

Color determination was carried out using a HANNA Honey colorimeter (HANNA C221 Honey Color Analyzer, Rhode Island, RI, USA), previously calibrated with glycerin (Glycerol HANNA instruments, Rhode Island, RI, USA), which gives the values in millimeters Pfund.

#### 2.4.4. Polyphenol and Flavonoid Content

The total phenolic content (TPC) was determined using an adapted Folin-Ciocalteu method [15]. Briefly, a honey solution for each sample was prepared (0.1 g/mL) and mixed with 10 mL of distilled water, 1 mL of Folin–Ciocalteu reagent and 4 mL of 7.5% sodium carbonate (Na_2_CO_3_) solution up to a final volume of 25 mL. After incubation at room temperature in dark for 1 h, the absorbance of the solution was measured by spectrophotometry at 765 nm. Gallic acid (GA) was chosen as a standard, using various concentrated solutions (0.01–0.50 mg/mL), and then a calibration curve was obtained. The results were expressed as gallic acid equivalents in mg/100 g of honey.

Total flavonoid content (TFC) was measured using an adaptation of the Dowd method [16]. The same first stock honey solution (0.1 g/mL) prepared for the determination of phenol content was used. This solution was dissolved until a concentration of 0.33 g/mL and 0.5 mL of 5% aluminum chloride (AlCl_3_) solution was added prior to incubation in dark for 30 min. The reaction yields a yellow color and their absorbance was determined spectrophotometrically at 425 nm after incubation in dark for 30 min. The TFC was calculated using a calibration curve with different quercetin solutions (0.002 to 0.01 mg/mL) and the results were expressed as mg equivalent of quercetin per 100 g of honey.

#### 2.4.5. Mineral Composition Analysis

Mineral content of honey was quantified by inductively coupled plasma mass spectrometry (ICP-MS) and by Atomic Absorption spectrometry (AAS) using a Spectrometer Varian SpectraAA-600 (Agilent Technologies, Santa Clara, CA, USA). First, the samples were warmed and sonicated to facilitate the homogenization of honey, then 5 mL of HNO_3_-H_2_O_2_ 9:2 were added to 0.5 g aliquots of the homogenized honey and afterwards were digested in a microwave oven (CEM MARSX press model) [17]. ASS was used to determine Na, K, Ca, Mg and Fe and ICP-MS for quantifying Mn, Cu, Zn, Cd, Pb and P. The results were expressed as mg per 100 g of honey.

### 2.5. Data Analysis

Sensorial data was analyzed using the XLSTAT Sensory tool by Addinsoft (Paris, France). This package let to obtain the characterization of the samples considering the attributes perceived by the 8 tasters. The procedure for sample characterization and panel evaluation was used. Significant statistical differences were set as *p*-value < 0.05. The radar charts for monofloral samples were done using the factorial values obtained in the characterization of products.

The package Analyzing data of XLSTAT was used as an exploratory statistical tool to reduce dimensionality of the multivariate data and to visualize them graphically, with minimal loss of information. PCA analysis was applied to identify groups of samples according to their botanical origin. This multivariate analysis allowed to summarize the information includes in the variables studied into a small number of principal components or factors providing a simplified interpretation of data variance through mathematical methods.

## 3. Results and Discussion

### 3.1. Sensorial Profile of Samples

**Figure 1:**
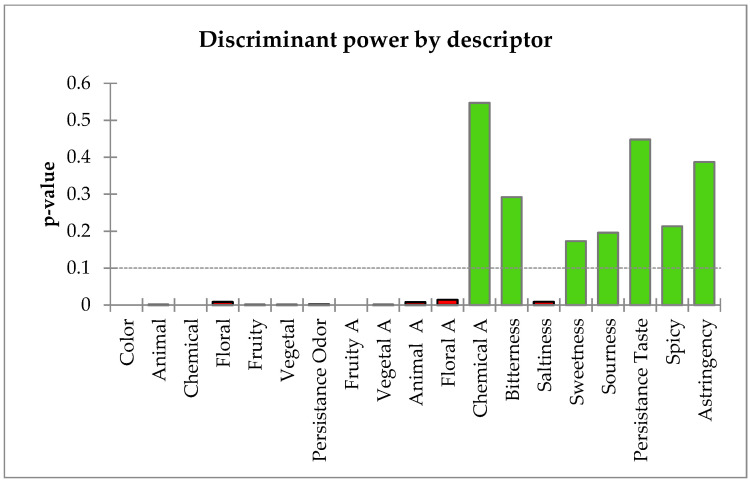
Sensorial descriptors used and their discriminant power.

Organoleptic properties are the first attributes that consumer can observed. These comprise visual properties as state, color, smell and aroma perceptions and taste. The descriptors considered in this work, to describe honey samples, had different discriminant power (Figure 1). The most useful were visual color, the different families of smell (animal, chemical, floral, fruity and vegetal), the persistence of smell, aroma perceptions (mainly fruity, vegetal, animal and floral families) and the saltiness for taste descriptors. The rest of the descriptors have been poorly detected so presented non-significant values.

All the samples were crystallized when the sensorial analysis was performed. Hence the scale used for color descriptors was from white to brown. The clearest samples were classified like straw color (two samples), 13 had gold color, five orange color and 10 brown color. The size of crystals was very fine or fine in 14 samples, medium in 15 samples while only one sample had large crystals. The tasters described the intensity of the smell as low being the predominant the vegetal in 21 samples, fruity in three and floral in two samples; however, secondary perceptions were mainly fruity, floral and in six samples, animal. The persistence of the odor was generally low or extremely low. Regarding the taste, besides the sweetness, tasters detected a degree of sourness and saltiness in most of the samples while bitterness was less common. When the aroma was considered, the most common perceptions were fruity, floral and in lesser extent vegetal. The most usual descriptors for vegetal smell or aroma were leaves, wood, mint, resin or cinnamon, for fruity were fresh fruit, tropical fruit, peach, dry grapes or fig, for floral were rose, lavender and violet and for animal leather and wax (except two samples in which tasters made associations with urine). Astringency was detected in a few samples. Finally, it should be mentioned that 15 samples had an intense smell to smoke (degraded family), that could be caused during the honey harvest. To protect quality beekeepers should improve honey management techniques.

### 3.2. Pollen Spectra and Main Botanical Origin of Samples

Ninety-six different pollen types were identified in the pollen spectrum of the honey samples including 77 nectariferous taxa and 19 taxa which do not produce nectar. Samples present a great diversity of pollen types, the mean value was 26, but 12 samples had more than 30 different pollen types. The botanical families Fabaceae, Asteraceae, Apiaceae and Lamiaceae provide the 36.0% of the pollen types, standing out the diversity of Fabaceae plants which were 15.3% of identified types. Within this family, *Genista* and *Hedysarum* pollen types were the most frequent pollen grains. The first was present in 96.7% of the samples and the second one in 83.3% of them.

*Genista* was dominant pollen in three samples and the maximum value reached was 73.9% whereas *Hedysarum* was dominant pollen in two samples and had a maximum value of 63.2%. For *Asteraceae* plants, *Galactites* t. was found in 70.0% but the values in the pollen spectra were lower than 7%, while *Aster* t., identified in 50.0% of honeys, had a maximum value of 39.7%. The most frequent Lamiaceae pollen was *Stachys* t., found in 70% of samples with a maximum value of 9.7%. Other common pollen type was *Thymus* t. (46.7% of samples) but values were always below 3%. Finally, Apiaceae plants mainly as *Foeniculum vulgare* t. and *Pimpinella anisum* t. were found in 73.3% and 50.0% honeys, respectively (Table 3).

Despite the frequency of the mentioned botanical families in the honey samples, some other plants highlighted in the pollen spectra of honeys. The first was *Erica* genus, represented by the species *E. arborea* and *E. multiflora*. This pollen type was found in 70.0% of samples and had a maximum value of 72.8%, being dominant pollen in four samples and secondary pollen in seven samples. In addition, Myrtaceae is well represented in the area due to the presence of *Eucalyptus*, used to reforestation, and *Myrtus* that grows in Mediterranean area commonly below 600 m, both are present as secondary pollen in samples. *Pistacia* is other representative Mediterranean taxa, was present in 73.3% of honeys frequently with values under 15% but was secondary pollen in two samples. The area of study had an important rainy period that contributes to facilitate the introduction of some forest species in mountain areas as *Castanea*. The genus was only found in two samples, but its high bee value merit is to be considered. In the case of Rosaceae plants, the stand out, *Rubus*, presented in 70% of samples as being in one dominant pollen. Another mention should be made to the presence of *Asparagus* pollen, with a value of 21.0% in one sample.

The honey samples had medium pollen content in quantitative terms. Values ranged from 1117 to 23,196 grains/g, with an average of 6710 grains/g. Most of the samples (66.7%) were classified in class II of Maurizio and 23.3% in class III. Only 10% of samples had low pollen content (Class I). This is according to the use of centrifugation to extract honeys from the combs.

### 3.3. Quality Evaluation of the Honey Samples

Overall, most of the honey samples showed acceptable quality parameters that were in accordance with the international legislation on honey quality [18].

The evaluation of the degree of freshness gave good results for most of the samples. HMF values were below the limit of 40 mg/kg, having a mean value of 9.0 mg/kg. The lowest value was 1.3 mg/kg and the highest value was 31.8 mg/kg. Regarding diastase content, samples had extremely low values with a mean value of 11.3 DI. The minimum diastase level was 4.8 DI and the maximum was 29.0 DI. Ten honey samples had a diastase activity below 8 DI (the minimum legal limit in international standards without considerations about HMF content).

One important parameter for honey stability is water content. It varied from 16.4% to 19.8%, with a mean value of 18.1%. Most of the samples were collected during July and August and only one in April, but there were no differences in water content in relation to the date of harvest. Electrical conductivity was in general medium with a mean value of 0.60 mS/cm, a minimum of 0.29 mS/cm and a maximum value of 1.35 mS/cm. For pH, the values oscillated from 3.5 to 4.4 with a mean value of 3.9. The values of electrical conductivity correspond mainly to blossom honeys; however, six samples presented values over 0.80 mS/cm. Regarding to free acidity, all samples had values below the maximum permitted in European legislation which is 50 meq/kg [18], ranging from 14.1 meq/kg to 46.5 meq/kg with a mean value of 32.4 meq/kg. According to Pfund scale, the color of samples varied from extra light amber (37 mm), to dark amber (135 mm) with a mean value of 77 mm. In addition to the differentiation between blossom honeys and honeydew honeys, the EC of honey is strongly related to its organic acids, ash content and proteins [19]. Therefore, the higher the content of the latter, the higher the obtained conductivity. A high content of organic acid and salts increases the free acidity present in honey.

#### 3.3.1. Mineral Content

Considering the average value of samples, the most abundant minerals were K followed by P, Na, Ca and Mg. The average value of K was 135.1 mg/100 g varying from a minimum of 43.1 mg/100 g to a maximum of 394.5 mg/100 g. P varied from 17.6 mg/100 g to 36.6 mg/100 g and had an average of 25.8 mg/100 g and Na, averaged 11.0 mg/100 g and ranged from 3.5 to 24.9 mg/100 g. Ca and Mg, had similar mean values of 7.5 mg/100 g and 5.5 mg/100 g, respectively, whereas Fe, Mn, Cu, Zn, were found with the lowest values ranging from the maximum value of 1.2 mg/100 g for Fe in some samples to values lower than 0.2 mg/100 g. Cd and Pb were always under the limit of detection. Normally, darker honeys had higher mineral content so heather honeys, chestnut honeys and honeydew honeys have the main values [20]. It has been proved by [21] that the botanical origin of honey has an impact on its elemental composition.

#### 3.3.2. Total Phenolic and Flavonoid Content

The phenol content (TPC) and flavonoid content (TFC) are other interested parameters in honeys. TPC values ranged from 41.8 mg GAE/100 g to 128.3 mg GAE/100 g, with a mean value of 70.6 mg GAE/100 g, while TFC were in accordance with TPC and ranged from 2.3 mg QE/100 to 9.7 mg QE/100 g, with a mean value of 4.9 mg QE/100 g.

These results were included within the interval found by [22] on honey samples from the same region and close to those reported by [23] on honeys from Algeria. Furthermore, TPC values were included in the range reported by [24] on Italian honey. Regarding TFC it is possible to find a great variation in values depending on the methodology used, but these values are higher than other reported from Moroccan honey [25] and Czech honey [26].

Both parameters are related with botanical origin of honey and hence with color, mineral content and electrical conductivity [20]. Previous studies have demonstrated a strong relationship between the phenolic profile of different honey types and their antioxidant capacities [20,27,28]. Therefore, further study of biological activities of honey produced in Babors Kabylia, such as the antioxidant power of honey samples, would be very interesting and necessary to show the correlation between the botanical origin and the content of phenolic compounds.

The most critical values for the quality of these samples correspond to water content and diastase index. All the analyzed samples presented a water content below the limit established by international legislation. However, values were higher than those reported for honeys from semiarid areas of Algeria [29]. Indeed, honeys produced in dry regions should have lower moisture content than honeys produced in humid regions. Similar values were reported by [7,30] on honeys produced in a nearby region, as well as those produced in the Mediterranean coast [6], coinciding with the humid area of Algeria. In any cases, the studied samples had relatively high-water content that should be considered due to their relationship with stability and conservation of honey. Furthermore, high quality honeys should contain low HMF content and high Diastase index. Both parameters are thermosensitive and increase with aging and prolonged heating of honey [31]. Diastase content was frequently lower than the stablished limit in international legislation; however, HMF content does not exceed the limit. Similar values to those found in these sample was reported by [31] for Algerian honeys but higher values were also found [7]. It is essential to remark that water content and diastase index are dependent on several factors, including botanical and geographical origin, environmental and seasonal conditions, the degree of maturity and the beekeepers’ handling during the honey harvest [32]. Studies on the common practices of honey management by beekeepers are necessary to determine the degree of influence on honey quality. In any case, the lack of professionalism combined with the quasi-total absence of training sessions related to beekeeping, hive maintenance, honey resources in the region and harvesting management, in addition to the propagation of Varroa disease and the absence of a legislative framework for quality recognition, can lead to problems linked to the quality of Algerian honey. In this context, to promote competitiveness with imported honey in the conventional market, it is recommended to promote knowledge of the characteristics of local honey and the participation of all stakeholders who can take actions to maintain the quality of the product.

### 3.4. Multivariate Analysis Applied to the Interpretation of Samples Characteristics and Typification of the Honey Samples

**Figure 2:**
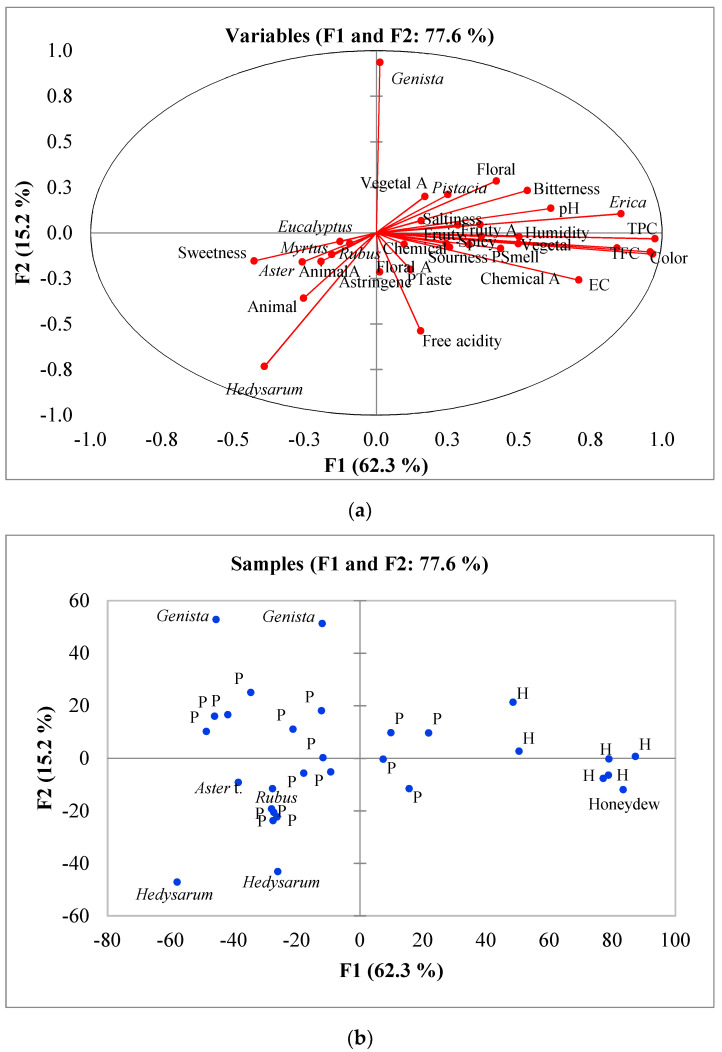
Principal Component Analysis (PCA). (**a**) Loading biplot of the variables included in the analysis, (**b**) Score biplot of the samples regarding component 1 and 2; P: Polyfloral, H: Heather.

Data on the sensorial analysis, the main pollen types in the pollen spectra of samples and some physico-chemical parameters were used for PCA analysis. In total, 34 standardized variables were introduced to create the covariance matrix. The five first factors explained 92.0% of the variance of the data, and the first two 77.6%. The first principal component (PC1) represented 62.3% of the variance and had the main correlation coefficients with the parameters of visual color (0.843), Pfund color (0.968), TPC (0.976), TFC (0.959), and *Erica* (0.856). The second one (15.2% of the variance) had the highest positive correlation coefficient with *Genista* (0.936) and negative with *Hedysarum* (−0.733). Some of the variables are highly correlated and appeared together in the biplot (Figure 2a). At the right, sensorial perceptions such as vegetal odor and aroma, saltiness, bitterness and sourness taste and persistence of taste and smell, pollen types such as *Erica* or *Pistacia* and electrical conductivity, color, TPC, TFC and PH. At the left, are animal smell and sweetness taste together the pollen types *Eucalyptus*, *Myrtus*, *Rubus*, *Aster* and *Hedysarum*.

In the biplot of the two first components, the cases are dispersed but some similar samples appeared close (Figure 2b). At the right, it can be seen a group of samples with reference H (heather samples) and one Hd (honeydew sample). Up are the samples with the highest content in *Genista* pollen type and down, at the left, are the samples with the highest content of *Hedysarum*, *Aster* and the sample with *Rubus* as dominant pollen. In the center, there are a group of samples classified as polyfloral honeys (P).

Analyzing the results, main taxa for honey production in this hotspot for biodiversity were *Erica*, *Hedysarum*, *Genista* plants and in lesser extent *Myrtus*, *Eucalyptus* and some Asteraceae plants. The importance of these plants for honey production was mentioned before by [4,33,34].

The shrubs occupy a large surface in deforested areas and slopes of the mountains. These Mediterranean maquis has a great interest for apiculture, being the most representative species *Erica arborea* developing mainly on nutrient-poor acidic soils and *Erica multiflora* growing in calcareous soils. Both have been found in samples and give recognized and high valued unifloral honeys. Other plants such as *Ceratonia siliqua* L., *Pistacia*, *Calicotome*, *Spartium junceum* L., and *Genista*, and some Lamiaceae like *Thymus* or *Lavandula*, *Phillyrea latifolia* L. or different species of *Cistus* and *Quercus* form plant communities with *Erica*, so their pollen appears in honeys samples. It is worth mentioning the controversy on the apicultural value of Genisteae plants; while some species are considered that do not produce nectar (such as *Calicotome spinosa*), others are considered good nectariferous source for honeys such as *Retama sphaerocarpa* [35] or *Spartocytisus supranubius* (L.f.) Christ ex G. Kunkel [36]. In the case of some *Genista* species, *Chamaecytisus* and *Retama* nectar secretion was reported [37]. Furthermore, through the pollen spectra of samples, the great diversity in pollen types can be observed, and further, the high number of pollen types by sample in accordance with biodiversity of the area. In this sense, melissopalynology is a key analysis with which to identify geographical origin of honeys and to determine melliferous species in the area where honey has been collected [38].

Mainly, the apiaries were in altitudes lower than 600 m above sea level and near to villages. In these areas, shrubs and spontaneous plants are most common; therefore, they predominate in the pollen spectra of honeys. Spontaneous species have high melliferous yield; among them, standing out are the Fabaceae plants such as *Hedysarum*, which is represented in the area for some species [39], many species of *Trifolium*, *Lotus* and other as *Securigera atlantica* Boiss and Reut., *Scorpiurus muricatus* L., *Tetragonolobus purpureus* Moench, *Astragalus echinatus* Murray. and *Medicago polymorpha* L. The importance of Fabaceae for honey production in the region was mentioned previously, either to produce monofloral honeys and/or to participate importantly in the production of polyfloral honeys [4,33,40,41,42]. Their importance also appears in studies carried out on honeys from the Mediterranean regions of Algeria [2,7,34,43].

Other important herbaceous plants for honey yield were Lamiaceae and Asteraceae; both are the most visited after Fabaceae [44]. The best represented pollen type in samples belonging to the family Lamiaceae was *Stachys*, which includes plants like *Stachys ocymastrum* or *Phlomis bovei* Noë and many other plants of this family; these are probably exclusives of the area. Something similar occurs with Asteraceae plants; the best represented pollen type was *Aster* t. with a pollen grain morphology similar for plants such as *Aster*, *Helichrysum stoechas* (L.) Moench, *Dittrichia viscosa* (L.) Greuter or *Phagnalon saxatile* (L.) *Cass.*, frequently occurring plants in the area. Other pollen types of Asteraceae were identified in samples in lesser extents, but due to the possible contribution of these plants to honey production and the fact that they normally appear underrepresented in the pollen spectra of honeys [35], a deep investigation about vegetation and plant distribution in the area could help in the identification of honeybee resources.

### 3.5. Characteristics of the Different Honey Types

The interpretation of the data allowed us to classify 12 samples, such as unifloral six heather honeys, two sulla (*Hedysarum*) honeys, two *Genista* honeys, one blackberry (*Rubus*) honey, one Asteraceae honey), one like honeydew honey and the rest, as polyfloral.

Table 4 shows the mean and standard deviation values for physicochemical and main pollen parameters. Polyfloral honeys showed the highest variation between samples in most of the variables as correspond to the heterogeneity of the group. A great variation in pollen content within polyfloral samples was noted, ranging from class I, II and III, although some heather honeys, *Hedysarum*, Asteraceae and honeydew were found to be in class III. Regarding color, polyfloral honeys were mainly light amber, as found in *Genista*, *Hedysarum*, Asteraceae and *Rubus* honeys. However, honeydew and heather honeys were the darkest honeys. The honeys presented similar water content that ranged from 16.8 to 19.6%. However, in terms of electrical conductivity and pH, honeydew honey presented the highest values. At the other extremes were *Genista* honey with the lowest electrical conductivity and *Rubus* honey with the lowest pH.

Although *Genista* honeys had the lowest free acidity, all honey types had similar values. The same occurred with the HMF content. Honeydew honey had the highest average value of diastase index, while the lowest level was observed in heather honeys. This difference could be explained by the fact that the concentration of diastase depends on several factors, including handling conditions, and could not be a reliable indicator of the honey’s origin. It could therefore be explained by the fact that the honeys may have been heated or improperly stored. The TPC and TFC also varied depending on the type of honey. Honeydew honey followed by heather honeys had the higher values of these compounds. Finally, the mineral content was higher in honeydew honey. Secondly, the high content of K, Ca, Mg and Fe in heather honeys were noteworthy. However, followed by Honeydew honey, the P content was higher in Asteraceae and *Hedysarum* honeys. *Rubus* honey type showed a light amber color but also a low TPC and TFC, although this type of honey showed a wide variation in color and varied from extra light amber to dark amber [45]. It is therefore necessary to study more *Rubus* honeys from this region to verify this finding.

**Figure 3:**
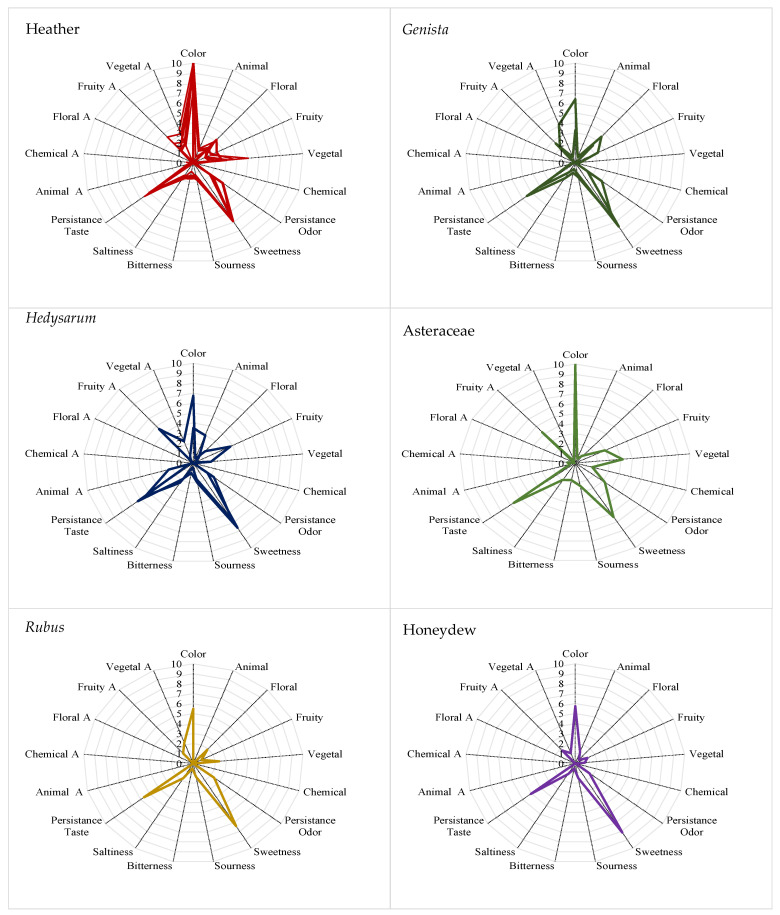
Chart diagrams for sensorial perceptions of monofloral honeys.

The sensorial profile of the different honey types can be seen in Figure 3. The six samples of heather honey presented a common pattern with dark color (brown), when crystallized, predominant vegetal smell (leaves and resin), some floral features and fruity (dry and tropical fruit). The persistence of the smell was low whereas the persistence of the taste was medium, and samples presented saltiness, bitterness and sourness in similar intensity. The aroma was mainly fruity and vegetal. The *Genista* samples had similar pattern in smell and aroma but different color, one had straw color and the other orange color (both were crystallized). The smell was mainly floral and in lesser extent fruity and aroma was mainly fruity. The persistence of smell was low, and the persistence of the taste was medium, the intensity of the sweetness was high and presented some saltiness and sourness notes. Sulla honeys were the most different regarding their sensorial profile, one was darker than the other and the smell and the aroma of one of them was animal (leather) while the other had floral and fruity smell. The persistence of the smell and the taste were similar in both cases. Asteraceae samples presented orange color, fruity smell and aroma with low intensity and persistence for smell, aroma and taste. Regarding the blackberry honey, highlighted the floral and fruity smell and the fruity aroma as well as the predominance of sweetness in taste. Finally, the honeydew sample had brown color, vegetal odor, fruity aroma and the highest persistence of smell.

The published scientific information about sensory profile of honey is scarce [12,35,46,47] and no information is available about sensorial properties of honey produced in Algerian country. The heather honeys were the most known and have similar sensorial profile to those previously described [35,48]. Regarding the sulla honeys were mainly described with floral smell but these samples were different in the smell having one of them fruity smell and the other animal smell, both with very low intensity [49]. Finally, the sensorial properties of Genista honeys are poorly described in literature, but its sensorial properties are near to other honey obtained from Fabaceae plants as *Spartocytisus supranubius* [36]. Other honey types are poorly known regarding their sensorial properties.

This is the first study focuses on the sensory properties of honey and their relationships with the botanical origin and the physicochemical properties of honeys from Babors Kabylia Region. Considering that this area is a hotspot for biodiversity and have a high potential for honey production, further studies could contribute to increase knowledge in the characteristics of the honey, the types that can be obtained and the most relevant plant species for honey yield and definitely contribute to the valorization of local beekeeping.

## 4. Conclusions

Honey samples collected from Babors Kabylia, which is an important biogeographical zone with great beekeeping potential, were analyzed to characterize the honey of this region using physico-chemical, melissopalynological and sensory parameters. The richness of the region in terms of melliferous plants has manifested through the production of a variety of monofloral honeys, including heather, *Genista*, sulla, Asteraceae and blackberry besides to the polyfloral honeys. Samples had a diverse sensory profile in terms of smell/flavor (vegetal, fruity and floral) highly appreciated by consumers. However, it is necessary to mention that some quality parameters related to hive management have been affected. It is therefore recommended to enhance knowledge on beekeeping management and to promote professional training for beekeepers to better manage the hive and improve both the quality and quantity of honey.

## Figures and Tables

**Table 1:** Geographical origin of honey samples and period of harvest.

Sample	Area	Harvest Period	Altitude (a.m.s.l.)
M01	Timsyet (Tizi N’berber)	Summer	312
M02	Draa El-Gaid	Summer	580
M03	Ighil Hassan	Summer	320
M04	Lota village	Summer	20
M05	Tahalaket (Tichy)	Summer	167
M06	Ijouyaze Sahel	Summer	205
M07	Adrar Oufarnou	Summer	280
M08	Ait Aissa, Aokas	Summer	185
M09	Djermana (Aokas)	Summer	250
M10	Agwni Oukouche (Melbou)	Summer	596
M11	Ouagaz Ichaabanen (Aokas)	Summer	245
M12	Annar Assam	Summer	335
M13	Annar Assam	Summer	335
M14	Timsyet (Tizi N’berber)	Summer	312
M15	Tahalaket	Spring	167
M16	Tizi N’berber	Summer	350
M17	Ouagaz Ichaabanen	Summer	245
M18	Mechta Ledjbel (Ziama)	Summer	590
M19	Draa El-Gaid	Summer	580
M20	Tabelout	Summer	305
M21	Tasabounet	Summer	220
M22	Zentout-Tamrijet	Summer	290
M23	Melbou	Summer	03
M24	Ait Idir-Adekar	Summer	500
M25	Tizi Ahmed	Summer	330
M26	Agounane-Derguina	Summer	300
M27	Djoa-Ajloh (Tichy)	Summer	260
M28	AiT Anane-Derguina	Summer	80
M29	Tababort	Summer	600
M30	Boukhlifa	Summer	280

**Table 2:** Main descriptors used for sensorial analysis.

Sensory Perception	Descriptors
Estate	Liquid, Crystallized
Color	White, Straw, Gold, Orange, Brown
Smell	Vegetal, Floral, Animal, Chemical, Fruity, Degraded
Taste	Sweetness, Bitterness, Saltiness, Sourness
Aroma	Vegetal, Floral, Animal, Chemical, Fruity, Degraded
Tertiary attributes	Astringency, Spicy

**Table 3:** Frequency classes of the main pollen types.

Family	Pollen Type	Max. (%)	D (≥45%)	A (45–15%)	R (15–3%)	I (3–1%)	Present ^1^
Amaryllidaceae	*Allium* t.	5.7	-	-	1	-	11
Anacardiaceae	*Pistacia*	30.0	-	2	7	4	22
Apiaceae	*Daucus carota* t.	3.9	-	-	2	2	7
Apiaceae	*Foeniculum vulgare* t.	9.0	-	-	5	6	22
Apiaceae	*Pimpinella anisum* t.	6.0	-	-	3	4	15
Asparagaceae	*Asparagus acutifolius*	21.0	-	1	-	-	1
Asteraceae	*Aster* t.	39.7	-	1	3	5	15
Asteraceae	*Galactites* t.	6.9	-	-	3	3	21
Boraginaceae	*Echium*	4.4	-	-	2	6	17
Capparaceae	*Capparis spinosa*	12.7	-	-	1	-	4
Cistaceae	*Cistus* t.	11.7	-	-	1	6	19
Cyperaceae	*Carex*	5.0	-	-	1	2	6
Ericaceace	*Erica*	72.8	4	7	7	4	25
Fabaceae	*Astragalus*	22.1	-	1	-	-	1
Fabaceae	*Ceratonia siliqua*	7.3	-	-	1	1	2
Fabaceae	*Genista* t	73.9	3	15	8	1	29
Fabaceae	*Hedysarum*	63.2	1	-	7	6	20
Fabaceae	*Lotus* t	7.3	-	-	4	5	21
Fabaceae	*Spartium junceum* t.	16.8	-	1	5	1	8
Fabaceae	*Trifolium repens* t.	17.9	-	1	10	4	26
Fagaceae	*Castanea*	17.1	-	1	1	-	2
Fagaceae	*Quercus*	7.6	-	-	2	2	12
Lamiaceae	*Stachys* t.	9.7	-	-	6	7	21
Lythraceae	*Punica granatum*	10.5	-	-	1	3	5
Myrtaceae	*Eucalyptus*	41.9	-	2	4	4	16
Myrtaceae	*Myrtus communis*	25.9	-	3	12	4	24
Oleaceae	*Olea europaea*	6.1	-	-	1	1	4
Rosaceae	*Crataegus* t.	7.1	-	-	4	2	8
Rosaceae	*Prunus* t.	4.9	-	-	2	1	12
Rosaceae	*Rubus*	58.7	1	1	3	7	21
Salicaceae	*Salix*	11.6	-	-	4	1	11
Tamaricaceae	*Tamarix*	5.7	-	-	1	1	4

^1^ Max.: maximum value reached by the pollen type in samples, D: number of samples in which the pollen type is dominant (percentage ≥ 45%), A: number of samples where the pollen type is between 45% and 15%, R: number of samples where the pollen type is between 15% and 3%, I: number of samples where the pollen type is between 3% and 1%, present: number of samples where the pollen type was identified. t.: pollen type common for different plant genera.

**Table 4:** Values (mean and standard deviation) of the studied variables considering the botanical origin of samples.

	Asteraceae	Rubus	Heather	Sulla	Genista	Honeydew	Polyfloral
	(*n* = 1)	(*n* = 1)	(*n* = 6)	(*n* = 2)	(*n* = 2)	(*n* = 1)	(*n* = 17)
Main Pollen (%)	*Aster t*	*Rubus*	*Erica*	*Hedysarum*	*Genista t*		
	39.8	58.7	54.2 ± 16.9	58.8 ± 6.2	72.0 ± 2.6		
N. Pollen Types	31	19	19 ± 4	19 ± 6	20 ± 2	18	27 ± 6
PK (pollen/g)	13686	5292	7248 ± 4192	11110 ± 11338	4174 ± 2982	14465	5118 ± 5440
Maurizio classes	III	II	II, III	III	II	III	I, II, III
Humidity (%)	16.8	19.2	18.4 ± 0.6	18.1 ± 2.4	17.9 ± 0.7	19.6	17.9 ± 1.0
EC (mS/cm)	0.57	0.82	0.75 ± 0.2	0.54 ± 0.3	0.33 ± 0.0	1.35	0.55 ± 0.2
pH	3.7	3.5	4.0 ± 0.2	3.6 ± 0.2	3.9 ± 0.0	4.4	3.8 ± 0.1
Free acidity	33.5	35.5	34.7 ± 10.2	39.0 ± 2.8	15.5 ± 0.7	38.5	32.8 ± 8.2
Color (mm Pfund)	52	58	122 ± 7	53 ± 23	53 ± 22	135	66 ± 17
	Light Amber	Light Amber	Dark Amber	Light Amber	Light Amber	Dark Amber	Light Amber
Diastase Index	8.5	7.6	7.5 ± 2.2	14.8 ± 2.8	8.6 ± 2.4	16.9	12.5 ± 5.9
HMF (mg/100 g)	10.3	10.5	4.9 ± 0.8	7.0 ± 5.2	4.1 ± 0.5	5.4	11.5 ± 7.9
Polyphenols (mg/100 g)	49.6	53.9	109.8 ± 16.4	52.6 ± 7.8	52.6 ± 11.2	128.3	60.5 ± 15.8
Flavonoids (mg/100 g)	3.3	3.6	7.9 ± 1.3	3.8 ± 1.6	3.2 ± 1.3	8.7	4.1 ± 1.3
Na (mg/100 g)	9.4	11.9	12.0 ± 4.6	9.1 ± 5.1	6.9 ± 1.9	9.8	11.5 ± 5.9
K (mg/100 g)	122.4	170.5	193 ± 60.5	105.5 ± 71.4	66.9 ± 33.6	394.4	109.5 ± 55.7
P (mg/100 g)	27.6	23.9	25.7 ± 1.8	26.6 ± 4.8	20.0 ± 3.4	36.1	25.8 ± 4.1
Ca (mg/100 g)	6.2	4.0	12.9 ± 4.8	6.2 ± 3.2	2.9 ± 0.7	14.7	6.1 ± 1.6
Mg (mg/100 g)	2.1	2.9	10.7 ± 3.5	2.7 ± 1.7	2.9 ± 1.8	11.2	4.3 ± 2.6
Fe (mg/100 g)	0.2	0.1	0.3 ± 0.2	0.3 ± 0.1	0.15 ± 0.1	1.3	0.15 ± 0.1
Mn (mg/100 g)	<0.2	<0.2	<0.35	<0.2	<0.2	0.4	<0.2
Cu (mg/100 g)	<0.2	<0.2	<0.2	<0.2	<0.2	<0.2	<0.2
Zn (mg/100 g)	0.1	0.1	0.1 ± 0.1	0.1 ± 0.0	0.1 ± 0.0	0.1	0.1 ± 0.1
Cd (mg/100 g)	<0.1	<0.1	<0.1	<0.1	<0.1	<0.1	<0.1
Pb (mg/100 g)	<0.1	<0.1	<0.1	<0.1	<0.1	<0.1	<0.1
